# Spatial and Verbal Routes to Number Comparison in Young Children

**DOI:** 10.3389/fpsyg.2018.00776

**Published:** 2018-05-24

**Authors:** Francesco Sella, Daniela Lucangeli, Marco Zorzi

**Affiliations:** ^1^Department of Experimental Psychology, University of Oxford, Oxford, United Kingdom; ^2^Department of Developmental Psychology and Socialisation, Università di Padova, Padova, Italy; ^3^Department of General Psychology, Università di Padova, Padova, Italy

**Keywords:** counting, numerical estimation, number line task, digit comparison, preschool children

## Abstract

The ability to compare the numerical magnitude of symbolic numbers represents a milestone in the development of numerical skills. However, it remains unclear how basic numerical abilities contribute to the understanding of symbolic magnitude and whether the impact of these abilities may vary when symbolic numbers are presented as number words (e.g., “six vs. eight”) vs. Arabic numbers (e.g., 6 vs. 8). In the present study on preschool children, we show that comparison of number words is related to cardinality knowledge whereas the comparison of Arabic digits is related to both cardinality knowledge and the ability to spatially map numbers. We conclude that comparison of symbolic numbers in preschool children relies on multiple numerical skills and representations, which can be differentially weighted depending on the presentation format. In particular, the spatial arrangement of digits on the number line seems to scaffold the development of a “spatial route” to understanding the exact magnitude of numerals.

## Introduction

A wealth of studies have established an intimate association between numbers and space (Hubbard et al., 2005; de Hevia et al., 2008; Nuerk et al., 2015; Patro et al., 2016). This association emerges early in development, as attested by the finding that 7 months-old infants display preferential looking for increasing numerical magnitude from left-to-right (De Hevia et al., 2014). Preschool children also associate small numerosities with the left side of space and large numerosities with the right side of space (Patro and Haman, 2012; see also, Patro et al., 2016). Interestingly, a spontaneous association between numerical quantity and space has also been found in new-born chicks (Rugani et al., 2015) as a sign of an evolutionarily ancient link.

Symbolic numbers are also strongly related to space as shown by the association between relatively small numbers with the left side of space and relatively large numbers with the right side of space (the SNARC effect; Dehaene et al., 1993). Patients with spatial neglect, who fail to pay attention to the left side of the visual field, also neglect small numbers when asked to verbally bisect numerical intervals (Zorzi et al., 2002). This has suggested that numerical magnitudes are mentally represented in a spatially ordered manner along a putative Mental Number Line (Restle, 1970; Dehaene et al., 1993; Zorzi et al., 2002) and that number processing involves orienting of attention in this “number space” (Zorzi et al., 2002, 2012; Fischer et al., 2003; Hubbard et al., 2005; Umiltà et al., 2009).

The ability to map symbolic numbers onto spatial positions has been extensively studied in developmental studies on primary school children using Siegler and Opfer’s (2003) number-to-position task (Siegler and Booth, 2004; Booth and Siegler, 2006; Siegler et al., 2009). More recently, Sella et al. (2017) observed that spatial mapping of symbolic numbers emerges during the early preschool period (also see, Berteletti et al., 2010) and appears to be crucial for understanding magnitude relationships for exact numbers. The aim of the present study was to further investigate how the ability to map numbers on a visual horizontal line is linked to symbolic number comparison skills.

Note that the understanding of symbolic numbers is typically linked to the development of counting. Around the age of two, toddlers begin to implement the counting routine to enumerate objects in their environment (Wynn, 1992). Children have to respect three foundational principles to achieve correct counting (Gelman and Gallistel, 1978): reciting the number words sequence in the established order (*stable order* principle); matching each object in the set to one and only one number word (*one-to-one correspondence* principle); identifying that the last number word represents the numerosity of the set (*cardinality* principle). Acquisition of counting principles is a long and error-prone process that engages children for about 1½ years, usually between 2 and 4 years of age (Sarnecka, 2015). According to the knower-level theory (Wynn, 1990; Carey, 2001; Sarnecka and Carey, 2008), children initially lack the understanding of number words: When requested to collect a certain number of objects (as in the Give-a-number task; Wynn, 1990), these children usually “grab” a handful of items without implementing any structured counting procedure. Subsequently, children sequentially learn the cardinal meaning of the number words from “one” to “four” and are able to provide numerosities from one to four when requested. These children are usually defined as Subset-knowers because their cardinal meaning of number words is limited to a subset of the counting list. Finally, children understand that the next number word in the counting list corresponds to one additional element in the counted set (i.e., *n* + 1, Gelman and Gallistel, 1978). Children at this stage can extend the cardinality principle to the entire counting list, thereby becoming Cardinal-Principle knowers (CP-knowers).

The acquisition of the cardinality principle should allow children to correctly map number words to corresponding objective external numerosities and, therefore, to understand the magnitude relation between number words (i.e., “eight is more than six”). Nevertheless, the acquisition of the cardinality principle does not imply a full understanding of the magnitude relation between number words. Indeed, some CP-knowers can fail in choosing the larger number when confronted with a pair of number words with magnitudes greater than 4 (e.g., 8 vs. 6), although they are successful when at least one number in the pair belongs to the small number range (≤4) (e.g., 4 vs. 2 or 6 vs. 3) (Le Corre, 2014). The paradox emerges from the fact that CP-knowers can reliably count both small and large numerical sets, as in the Give-a-number task, thereby showing the ability to connect number words to the corresponding external numerical quantities. Le Corre (2014) observed that the ability to compare pairs of large number words was present only in a subset of CP-knowers who were also able to reliably estimate large (i.e., >4 items) briefly visually presented numerosities (i.e., numerosity estimation). These children were referred to as CP-mappers, because their ability to map external numerosities onto number words is not derived by merely implementing the counting routine. Accordingly, these children know that later number words in the counting list are associated with larger numerical quantities (i.e., later-greater principle) and, then use this knowledge to determine the larger between two number words.

Sella et al. (2017) used a similar approach to investigate the relation between the acquisition of the cardinality principle and spatial mapping of numbers in a sample of preschool children. CP-knowers were classified as mappers when they could reliably place numerals on the horizontal visual line in the number-to-position task (1–10 interval) and as non-mappers when their positioning lacked any numerical meaning (e.g., all the numbers placed in the middle of the line). Crucially, only CP-mappers proficiently chose the larger between two visually presented Arabic digits whereas CP-non-mappers’ performance was close to chance level. Note that the spatial arrangement of digits on the number line is a powerful source of information because the magnitude of a digit can be conveyed by its location in relation to the location of other digits. Children who have internalized the spatial disposition of digits and understood that spatial shifts along the line represent changes in magnitude (*spatial mapping principle*; Sella et al., 2017) can use this information to infer the magnitude of numerals and compare them.

In summary, the magnitude comparison of number words seems to relate to the ability to map external numerical quantities onto the counting list (Le Corre, 2014), whereas the ability to compare visually presented digits may be linked to the ability to spatially map numbers (Sella et al., 2017). In the case of number words, the ability to linearly map external numerosities to the counting list marks the understanding that the later number words in the counting list are associated with larger numerical quantities. For Arabic digits, instead, the ability to map them to space informs about the magnitude of digits based on their absolute position on the line and their relative position compared to other digits. However, it remains unclear whether the contribution of numerosity estimation and spatial mapping are tied to a specific presentation format or are both related to the understanding of the magnitude relation between symbolic numbers.

More broadly, the investigation of format-dependent acquisition of the numerical meaning of symbols in young children is rather sparse. Some authors have suggested that children independently associate number words and Arabic digits to the corresponding numerical quantities and later number words are mapped to Arabic digits (Benoit et al., 2013). Others, instead, have suggested that the mapping between number words and Arabic digits is learnt after the mapping between number words and numerical quantities (Hurst et al., 2016). Interestingly, CP-knowers fail in transferring the cardinality knowledge of number words to Arabic digits, even though they can correctly read Arabic digits, thereby converting them from the visual to the verbal format (Knudsen et al., 2015). A recent detailed investigation of the mapping between number words, Arabic digits, and numerical quantities highlighted that the mapping between digits and numerical quantities contributed to the digit comparison performance, with an indirect contribution of the word-digit and word-quantity mappings (Jiménez Lira et al., 2017). Overall, these results suggest the existence of a separate (visual) route for learning the numerical meaning of Arabic digits, which coexists in parallel to the learning of the numerical meaning of number words. Nevertheless, it is still plausible that children initially learn the numerical meaning of number words and subsequently transfer this knowledge to Arabic digits while learning to read them.

The aim of the present within-subjects study was to investigate this issue in relation to children’s ability to compare number words and Arabic digits. Assessing whether a core numerical skill, like symbolic number comparison, is modulated by the presentation format can inform theories of numeracy development and might have an impact on educational practices. Our hypothesis that symbolic number comparison in young children relies on distinct routes (spatial vs. verbal) depending on the presentation format leads to specific predictions. That is, performance in number words comparison should be related to the ability to estimate large numerical quantities (Le Corre, 2014) after controlling for cardinality knowledge, whereas accuracy of spatial mapping should be irrelevant. Conversely, performance in Arabic digit comparison should be related to the accuracy of spatial mapping after controlling for cardinality knowledge (Sella et al., 2017), whereas the precision in estimating large numerical quantities should be irrelevant. It is worth considering that children may transform the Arabic digit comparison into a number words comparison by transcoding the Arabic code into verbal code (Dehaene, 1992). If that is the case, the ability to read digits and performance in the number words comparison task should explain the performance in the Arabic digit comparison task and the role of the accuracy in spatial mapping should be minimal.

## Materials and Methods

### Participants

Sixty preschool children from a school located in north-eastern Italy took part in the experiment after informed consent was obtained from parents or legal guardians. Seven children were removed from the analyses because they failed to complete the experimental session (three children interrupted the session and one child provided only three estimates in the numerosity estimation task) or they had a cognitive disability as reported by the teachers (three children). Six additional participants were removed from analyses because they failed to correctly recite the numerical sequence at least up to 10 in the forward enumeration task (see below), which was a crucial requirement to perform the numerosity estimation task (which contained trials with numerosity up to 10). The final sample was composed of 47 children (17 boys, *M*_age-in-months_ = 64, *SD* = 9, range = 43–79), a sample size that is in line with those of the relevant previous studies (Le Corre, 2014; Sella et al., 2017).

### Procedure

Children were met individually in a separate quiet room during school hours and completed all the tasks in one experimental session (approximately 20–30 min depending on the child’s ability). Children completed the numerical tasks in the following order: forward enumeration, backward enumeration, give-a-number, naming, number line, Arabic digit comparison, number words comparison and numerosity estimation. Children were allowed to take a break between tasks and they could interrupt the experimental session at any time. The results from the backward enumeration task are not reported in the present study.

### Numerical Tasks

#### Forward Enumeration

Children were asked to recite the numerical sequence starting from one and were stopped when they reached 50 or when they could not go any further. Children could correct themselves immediately if they realized they have committed a mistake. The experimenter did not provide any feedback. This task was administered to ensure that children were at least able to recite the counting list up to 10, which was the largest numerosity presented in the numerosity estimation task (see below).

#### Give-a-Number (GaN)

A small basket with 15 wooden tomatoes (approximately 3 cm of diameter) was at the child’s disposal before starting the task in order to familiarize the child with the materials. The task was introduced as a role-play game in which the experimenter played the role of a customer and the child played the role of the grocer. The experimenter said: “*Let’s play the market game! You are a grocer and I’m a customer that wants to buy some delicious tomatoes. Ok? Are you ready?*” The experimenter then said: “*Hello! May I have* n *tomato/es, please?*” As soon as the child gave the selected number of tomatoes, the experimenter said: “*Is this/Are these* n *tomato/es?*” The child was allowed to modify the number of tomatoes until she was sure about the number. The experimenter asked for 1, 2, 3, 4, 5, 8, and 10 tomatoes in random order and the percentage of correct responses was calculated.

#### Naming

Children were presented with an Arabic digit in the center of the computer screen and were asked to name it aloud. Numbers from 0 to 20 were presented randomly. Only digits from 1 to 9 were considered given that the same range of digits was presented in the Arabic digit comparison task, in which children were presented with digits that were not read by the experimenter. One point was awarded for each correct naming and the percentage of correct responses was calculated.

#### Number Lines 1–10 (NL)

A black horizontal line, with no tick marks, was presented in the middle of the computer screen with the number one (“1”) placed just below the left-end of the line and the number ten (“10”) placed just below the right-end. The number to be positioned (e.g., “4”) was presented inside a box in the upper left corner of the screen. For every trial, the experimenter said: “*This line goes from one to ten* [pointing at the numbers]. *Where is the correct place for* n [pointing at the number in the upper left corner]? *Show me the correct place moving the mouse and pressing the mouse button when you are on the right place!*” Children placed the numbers on the line by moving an arrow using the mouse and clicking the mouse button to confirm the selected position. The movement of the arrow was constrained to the horizontal line to facilitate the response. After pressing the mouse button, a red dot appeared on the selected location. There were two training trials (i.e., 1 and 10) in which, if the positioning of the target number was not accurate, the experimenter indicated to the child the correct position. The experimenter intervened only 4 times to correct children in the training trials. Out of 47 children included in the study, 44 correctly placed the number 1, one child placed 1 close to the position of 2 and two children placed 1 almost in the position of 10. Forty-six children correctly placed the number 10 and one child placed it close to the position of 9. After the training trials, children had to place eight randomly presented numbers (i.e., 2, 3, 4, 5, 6, 7, 8, and 9), three times each for a total of 24 trials. For each child, we calculated the mean percentage of absolute error (PAE) as follows: (|estimate-target number|/9)^∗^100. We also calculated the individual regression slope of estimates as function of target numbers (*M* = 0.69, *SD* = 0.45, range: -0.40, 1.27): children with a positive and significant regression slope were classified as *spatial mappers* (*n* = 34; *M* = 0.92, *SD* = 0.25, range: 0.25, 1.27) whereas the remaining children were classified as *non-mappers* (*n* = 13; *M* = 0.09, *SD* = 0.25, range: -0.40, 0.39).

#### Number Comparison

##### Number words comparison (adapted from Le Corre, 2014)

Two gray boxes were horizontally presented in the lowest part of the computer screen. Then, the experimenter read the text written above the boxes: “*In this box* [pointing the box on the left side] *there is/are* n *ball/s and in this box* [pointing the box on the right side] *there is/are* m *ball/s*. *Which box has more balls?*” The child responded by pointing the box (or simply saying which was the largest number) and the experimenter recorded the response by pressing the left or right button of the touchpad. After the response, the two boxes were replaced by two images showing the actual numerosities. The images representing the comparison numerosities were generated following a method to control for the influence of physical variables (e.g., cumulative surface area, convex hull; Gebuis and Reynvoet, 2011). Then, the experimenter read the text written above the boxes: “*This box* [pointing the box on the left side] *contained* n *ball/s and this box* [pointing the box on the right side] *contained /is* m *ball/s*.” The numbers read by the experimenter were written in the verbal format (e.g., “four”). There were twelve randomly presented comparisons (i.e., 1–2, 1–4, 1–6, 1–8, 2–3, 2–9, 3–6, 3–8, 4–9, 6–7, 6–9, 8–9) repeated twice to have the larger number in both locations. For each participant, we calculated the percentage of correct responses as main performance index.

#### Arabic Digit Comparison

Two digits were horizontally presented, respectively, on the left and right side of the computer screen. The child was asked to indicate the side of the larger digit by pressing the corresponding (left or right) touchpad button. There were 72 randomly presented trials displaying all possible pairs of digits from 1 to 9 twice. The larger number was equally presented in both locations. We calculated the percentage of correct responses as accuracy measure.

#### Numerosity Estimation

Children verbally estimated the numerosity of a set composed of black squares presented in the center of the screen for 1 s. There were two practice trials (i.e., 2 and 8) and then the numerosities 1, 2, 3, 4, 6, 8, and 10 were randomly displayed four times for a total of 28 trials. For each target numerosity, in half of the sets the item size diminished with increasing numerosity (i.e., equal cumulative surface area) whereas in the other half the item size was constant (i.e., constant item size). We manipulated item size to prevent children from basing their numerical estimates on visual cues instead of focusing on the numerosity of the presented sets. For each participant, we calculated the mean absolute deviation between the estimate and the target number separately for small (≤4) and large (>4) target numerosities. We also computed the individual regression slopes of the estimates as function of target numerosities from 6 to 10 (*M* = 0.42, *SD* = 0.49, range:-0.88, 1.31). Following Le Corre and Carey (2007)’s classification, children displaying a slope ≥ 0.3 were classified as *verbal mappers* (*n* = 30) whereas other children were classified as *non-mappers* (*n* = 17).

## Results

Statistical analyses were conducted using the free software R (R Core Team, 2016) with the following packages: BayesFactor, using default priors (Morey and Rouder, 2015); Hmisc (Harrell et al., 2016); psych (Revelle, 2016); xlsx (Dragulescu, 2014); Rmisc (Hope, 2013); lmSupport (Curtin, 2016); plyr (Wickham, 2011); metafor (Viechtbauer, 2010); car (Fox and Weisberg, 2011); lmtest (Zeileis and Hothorn, 2002); Reshape2 (Wickham, 2007). We report Bayes factors (BF_10_) expressing the probability of the data given H1 relative to H0 (i.e., values larger than 1 are in favor of H1, the alternative hypothesis, whereas values smaller than 1 are in favor of H0, the null hypothesis). When comparing regression models, we report the Bayes factors (BF) as the ratio of BFs_10_ between compared models. If the ratio between BF_10_ of model A and BF_10_ of model B is larger than 1, then there is evidence for model A. Conversely, if the ratio is smaller than one there is evidence for model B. We describe the evidence associated with BFs as “anecdotal” (1/3 < BF < 3), “moderate” (BF < 1/3 or BF > 3), “strong” (BF < 1/10 or BF > 10), “very strong” (BF < 1/30 or BF > 30), and “extreme” (BF < 1/100 or BF > 100) (Jeffreys, 1961; Wagenmakers et al., 2016, 2017). Data and code can be found at https://osf.io/swg8r/?view_only=0fa72144bc1046c99efc0ee258ccf2b9.

We removed those trials with response time below 200 ms (i.e., anticipation) in the computerized tasks: this applied to only one trial in the Arabic digit comparison task. In the numerosity estimation task, we removed absent responses (e.g., “I don’t know”; 3 trials) and trials with estimates above 20 (extreme responses; 23 trials). We ran a Bayesian repeated measures ANOVA on the mean estimate with Target numerosity [1, 2, 3, 4, 6, 8, and 10] and Stimulus set [equal cumulative surface area, constant item size] as within-subjects factors. The model with only Target numerosity yielded the largest evidence compared to the null model (BF_10_ = 6.39 × 10^196^) and it was superior to the model also including Stimulus set (BF_10_ = 8.76 × 10^195^) or the model including the interaction between Target numerosity and Stimulus set (BF_10_ = 1.31 × 10^194^). This ensured that the estimates did not vary depending on the visual properties of the presented numerical sets (i.e., equal cumulative surface area and constant item size).

The main descriptive statistics of the administered tasks are reported in **Table 1**.

**Table 1:** Descriptive statistics for the administered numerical tasks.

Task	*M*	*SD*	95% CI
Naming (1–9) (% of correct responses)	79	30	[70–88]
Give-a-Number (% of correct responses)	88	21	[81–94]
NL task (PAE)	20	12	[16–23]
Numerosities estimation (absolute difference)			
- Small numerosities (≤4)	0.22	0.3	[0.13–0.3]
- Large numerosities (>4)	2.09	0.89	[1.82–2.35]
Arabic digit comparison (% of correct responses)	82	19	[77–88]
Number words comparison (% of correct responses)	86	14	[82–91]

### Regression Analyses

We ran two separate regression analyses in order to specifically highlight the contribution of the assessed numerical skills to number words and Arabic digit comparison, respectively. For all the regression models reported in **Tables 2**, **3**: residuals were normally distributed (non-significant Shapiro tests, except for Model 1, *p* = 0.006, in **Table 2**; Model 1, *p* < 0.001, and Model 3, *p* = 0.017, in **Table 3**); multicollinearity was absent (i.e., all Variance inflation Factors were lower than 4 for the models with two or more predictors); heteroscedasticity was absent (i.e., non-significant Breusch–Pagan tests, except for Model 1, *p* = 0.05, in **Table 2**); no influential observations were found (i.e., all Cook’s distances were below or equal 1).

**Table 2:** Summary of the regression models with proportion of correct responses (arcsine transformed) in the number word comparison task as outcome variable.

Model	Measures	*B*	95% CI	Bayes factor (BF_10_) for the comparison with the null model	*R*^2^	Bayes factor for model comparison
1	GaN (% correct)	0.006	[0.003 0.009]	144	0.27	
2	GaN (% correct)	0.004	[0.001 0.008]	107	0.31	BF_10_ model 2/BF_10_ model 1 = 0.74
	NL (PAE)	-0.005	[-0.011 0.002]			
3	GaN (% correct)	0.005	[0.002 0.008]	160	0.32	BF_10_ model 3/BF_10_ model 1 = 1.11
	Numerosity estimation large numerosities (absolute difference)	-0.066	[-0.139 0.007]			
4	GaN (% correct)	0.004	[0.0003 0.0075]	91	0.35	BF_10_ model 4/BF_10_ model 1 = 0.63
	NL (PAE)	-0.004	[-0.010 0.003]			
	Numerosity estimation large numerosities (absolute difference)	-0.057	[-0.131 0.018]			

**Table 3:** Summary of the regression models with proportion of correct responses (arcsine transformed) in the Arabic digit comparison task as outcome variable.

Model	Measures	*B*	95% CI	Bayes factor (BF_10_) for the comparison with the null model	*R*^2^	Bayes factor for model comparison
1	GaN (% correct)	0.007	[0.004 0.011]	395	0.31	
2	GaN (% correct)	0.004	[-0.0001 0.007]	14338	0.46	BF_10_ model 2/BF_10_ model 1 = 36
	NL (PAE)	-0.011	[-0.018 -0.005]			
3	GaN (% correct)	0.006	[0.003 0.009]	674	0.37	BF_10_ model 3/BF_10_ model 1 = 1.7
	Numerosity estimation large numerosities (absolute difference)	-0.085	[-0.167 -0.004]			
4	GaN (% correct)	0.003	[-0.001 0.007]	10731	0.49	BF_10_ model 4/BF_10_ model 2 = 0.75
	NL (PAE)	-0.010	[-0.017 -0.004]			
	Numerosity estimation large numerosities (absolute difference)	-0.059	[-0.135 0.016]			
5	GaN (% correct)	0.001	[-0.002 0.004]	4.11 × 10^7^	0.66	BF_10_ model 5/BF_10_ model 1 = 104155
	Naming (% correct)	0.002	[-0.0004 0.004]			
	Number words comparison (% correct)	0.013	[0.008 0.017]			
6	GaN (% correct)	0.001	[-0.003 0.004]	2.3 × 10^8^	0.72	BF_10_ model 6/BF_10_ model 5 = 5.61
	Naming (% correct)	-0.001	[-0.003 0.002]			
	Number words comparison (% correct)	0.012	[0.008 0.016]			
	NL (PAE)	-0.009	[-0.015 -0.002]			

#### Number Words Comparison

In the first regression analysis, we used the proportion tion of correct responses in the number words comparison task (transformed with arcsine square root formula^1^; Osborne, 2010) as the outcome variable (**Table 2**). There was extreme evidence for the model including the accuracy in the GaN task (Model 1). Compared to Model 1, there was anecdotal evidence for the models also including PAE in the NL task (Model 2), the absolute deviation for larger numerosities from the numerosity estimation task (Model 3), and all three predictors together (Model 4). We replaced the absolute difference in the numerosity estimation task with a variable coding for the status of verbal mapper (=1) and non-mapper (=0) as proposed by Le Corre and Carey (2007). There was moderate evidence against the model including the status of mapper compared to the model with only GaN performance (BF = 0.30), also when we considered the number word comparison accuracy only for large number words (>4; BF = 0.34) as in study Le Corre’s (2014). Accordingly, verbal mappers and non-mappers displayed a similar accuracy when comparing all number words (Verbal mappers: *M* = 87%, *SD* = 14; Verbal non-mappers: *M* = 85%, *SD* = 14; Bayesian *t*-test: BF_10_ = 0.32, moderate evidence) and only large number words (Verbal mappers: *M* = 79%, *SD* = 22; Verbal non-mappers: *M* = 79%, *SD* = 17; Bayesian *t*-test: BF_10_ = 0.30, moderate evidence). The same pattern of results emerged when we compared the model with only GaN accuracy with the model also including the linear slope for large numerosities in the numerical estimation task as predictor of all number words comparison (BF = 0.28) and large number words comparison (BF = 0.33). Finally, there was moderate evidence against the model including age in months and the performance in the GaN compared to the model with only GaN accuracy (BF = 0.28), thereby confirming the predominant role of cardinality knowledge. Overall, the results strongly support the relation between cardinality knowledge and number words comparison accuracy, whereas there was no clear evidence for a role of numerosity estimation and spatial mapping abilities.

#### Arabic Digit Comparison

In the second regression analysis, we used the proportion of correct responses (transformed with arcsine square root formula) in the Arabic digit comparison task as the outcome variable (**Table 3**). There was very strong evidence for the model including GaN accuracy and the PAE in the NL task (Model 2) compared to the model including only the accuracy in the GaN task (Model 1). Conversely, there was anecdotal evidence for the model including the accuracy in the GaN task and numerosity estimation for large numerosities (Model 3) compared to the model with only the accuracy in the GaN task (Model 1). Similarly, there was anecdotal evidence for the model (Model 4) including the absolute deviation for large numerosities in the numerosity estimation task compared to the model including the accuracy in the GaN task and the PAE in the NL task (Model 2).

We also assessed whether performance in the Arabic digit comparison task could be fully accounted for by the ability to compare number words and the accuracy in naming Arabic digits, thereby excluding the influence of spatial mapping. Therefore, in Model 5, we simultaneously included the accuracy in the GaN task, the accuracy in the naming task, and the accuracy in the number words comparison task. There was extreme evidence for the inclusion of the accuracy in naming and in the comparison of number words (Model 5) compared to the model with only the accuracy in the GaN task (Model 1). In Model 6, we also entered the PAE in the NL task. We found moderate evidence for the model also including the PAE in the NL task, thereby suggesting a specific contribution of spatial mapping to the understanding of magnitude relation between Arabic digits (Sella et al., 2017). Accordingly, spatial mappers were more accurate in comparing Arabic digits compared to non-mappers (Spatial mappers: *M* = 90%, *SD* = 14; Spatial non-mappers: *M* = 60%, *SD* = 13; Bayesian *t*-test: BF_10_ = 403543).

The absolute difference for small numerosities in the numerosity estimation task was never a relevant predictor when entered in the previous regression models (all BFs < 1).

## Discussion

In the present study, we investigated the specific role of numerosity estimation and spatial mapping of numbers in the ability to compare auditorily presented number words and visually presented Arabic digits. Previous studies suggested that the ability to compare number words might be associated with numerosity estimation after controlling for cardinality knowledge (Le Corre, 2014). Similarly, the comparison of Arabic digits has been related to the ability to spatially map numbers on the visual line (Sella et al., 2017). Here, the comparison of number words related to cardinality knowledge but not to numerical estimation or spatial mapping accuracy. Children who knew that later number words in the counting list are associated with larger numerical quantities (i.e., verbal mappers) or were more accurate in mapping numbers on the visual line did not show a better performance in choosing the larger between two number words. Conversely, the ability to spatially map numbers strongly related to the comparison of visually presented Arabic digits. Crucially, we found moderate evidence for the relation between spatial mapping and Arabic number comparison even after controlling for the accuracy in reading Arabic digits and comparing number words, thereby addressing the potential caveat that the task might be transformed into verbal comparison after transcoding the digits into number words. These results suggest that the comparison of Arabic digits entails a specific spatial component that is captured by the accuracy of spatial mapping. In this regard, we have previously suggested that the spatial arrangement of digits along the line may scaffold the representation of exact numerical magnitude (Sella et al., 2017).

Previous studies have shown that the acquisition of the cardinality principle does not imply a mapping between exact magnitude and number words but rather entails the understanding that the last recited number word denotes the cardinality of the counted set (Davidson et al., 2012; Le Corre, 2014). This view is supported by the finding that some children who have acquired the cardinality principle, as measured by the GaN task, fail in choosing the larger between two number words within their counting range (Le Corre, 2014). It has been proposed that the understanding that later number words in the counting list are associated with large numerical quantities (i.e., the later-greater principle), or, more broadly, the precision of numerosity estimation (i.e., ANS-to-word mapping) might lead children to infer the numerical magnitude of number words. Nevertheless, this view was neither supported nor discarded by the results of the present study. A replication with a larger sample size would disentangle whether the estimation of large numerical quantities is actually related to the ability to compare number words. Conversely, spatial mapping ability was clearly related to understanding the exact magnitude of numerals, as measured by the Arabic digit comparison task. In this vein, the numerical magnitude of a digit can be conveyed by its spatial position on the line and with respect to the positions of other digits, conceivably through a symbol-to-symbol relation (Nieder, 2005; Vogel et al., 2014; Reynvoet and Sasanguie, 2016). The correlational nature of our study prevents us from inferring any casual direction between spatial mapping of numbers and magnitude understanding. Nevertheless, there is evidence that training spatial mapping of numbers leads to better performance in comparing Arabic digits (Siegler and Ramani, 2009; Ramani et al., 2012), thereby supporting the role of the “spatial mapping principle” (Sella et al., 2017) in the acquisition of exact numerical meaning of symbolic numbers.

The results of the present study suggest that children rely on multiple numerical skills and representations, which are differently weighted depending on numerical format. The presentation format plausibly leads children to rely on distinctive representations and strategies when comparing symbolic numbers. In the case of number words, the verbal format might lead children to rely on a verbal mechanism, such as counting. In this regard, it is worth noting that the comparison of each pair of number words was followed by the presentation of the corresponding numerosities in the current experimental paradigm (following Le Corre, 2014). In addition to providing visual feedback on the choice, this is likely to have trigged an enumeration strategy, even though children were not allowed to count the elements in the set but were moved immediately to the next trial. Conversely, the visual presentation of Arabic digits may have triggered a “number line” representation to choose the larger digit based on its spatial position.

More broadly, young children progressively integrate multiple representations of numerical information (verbal, visual, and analogical) and learn to switch from one representation to another (Dehaene and Cohen, 1995; Kucian and Kaufmann, 2009). It has been suggested that children first map number words to numerical sets, then map Arabic digits to numerical sets, and finally associate number words to Arabic digits (Benoit et al., 2013). Conversely, others have found that children first create an association between number words and the corresponding numerical quantities, then associate number words to Arabic numerals (Hurst et al., 2016). Similarly, preschool children first learn the cardinal meaning of number words, then to read Arabic digits, and finally learn the cardinal meaning of numerals and how to order them (Knudsen et al., 2015). Overall, this reveals a complex scenario in which children build connections between different representations of numbers in a relatively short time window. The integration of different representations of numbers is likely to be heavily influenced by individual experience that children have with numbers. For example, Arabic numerals might be introduced at different times across a sample of preschool children, which would clearly affect their understanding of their numerical meaning. Therefore, it would not be surprising to observe variability and divergent developmental patterns across different studies. Future research may describe the different developmental patterns associated with the integration of multiple representations of numbers and highlight the more efficient ways to achieve full understanding of the magnitudes associated with symbolic numbers. This kind of evidence would be extremely valuable for cognitive scientists and for educators interested in improving children’s early numerical skills.

## Conclusion

Preschool children use different numerical skills and representations depending on the presentation format to compare the numerical magnitude of symbolic numbers. The results of the present study suggest that the comparison of number words relates to cardinality knowledge whereas the comparison of Arabic numerals specifically relates to the spatial mapping of numbers. This finding supports the hypothesis that a spatial mapping principle scaffolds the acquisition of symbolic number knowledge.

## Ethics Statement

This study was carried out in accordance with the recommendations of the national ethics guidelines for psychologists [Codice Deontologico degli Psicologi Italiani] with written informed consent obtained from parents or legal guardians. All parents or legal guardians gave written informed consent in accordance with the Declaration of Helsinki. The protocol was approved by the Ethics Committee for Psychology Research of the University of Padova.

## Author Contributions

FS, DL and MZ designed the study. FS supervised data collection, performed statistical analyses, and drafted the manuscript. DL and MZ provided critical revisions of the article. All authors approved the final version of the manuscript.

## Conflict of Interest Statement

The authors declare that the research was conducted in the absence of any commercial or financial relationships that could be construed as a potential conflict of interest.

## Footnotes

1 The same pattern of results emerged when regression models were run on the proportion of correct responses in the number words comparison task and in the Arabic digit comparison task.
